# Natural Extracellular Electron Transfer Between Semiconducting Minerals and Electroactive Bacterial Communities Occurred on the Rock Varnish

**DOI:** 10.3389/fmicb.2019.00293

**Published:** 2019-03-04

**Authors:** Guiping Ren, Yingchun Yan, Yong Nie, Anhuai Lu, Xiaolei Wu, Yan Li, Changqiu Wang, Hongrui Ding

**Affiliations:** ^1^The Key Laboratory of Orogenic Belts and Crustal Evolution, Beijing Key Laboratory of Mineral Environmental Function, School of Earth and Space Sciences, Peking University, Beijing, China; ^2^College of Engineering, Peking University, Beijing, China

**Keywords:** bacterial communities, semiconducting minerals, varnish, extracellular electron transfer, light

## Abstract

Rock varnish is a thin coating enriched with manganese (Mn) and iron (Fe) oxides. The mineral composition and formation of rock varnish elicit considerable attention from geologists and microbiologists. However, limited research has been devoted to the semiconducting properties of these Fe/Mn oxides in varnish and relatively little attention is paid to the mineral–microbe interaction under sunlight. In this study, the mineral composition and the bacterial communities on varnish from the Gobi Desert in Xinjiang, China were analyzed. Results of principal components analysis and *t*-test indicated that more electroactive genera such as *Acinetobacter*, *Staphylococcus*, *Dietzia*, and *Pseudomonas* gathered on varnish bacterial communities than on substrate rock and surrounding soils. We then explored the culture of varnish, substrate and soil samples in media and the extracellular electron transfer (EET) between bacterial communities and mineral electrodes under light/dark conditions for the first time. Orthogonal electrochemical experiments demonstrated that the most remarkable photocurrent density of 6.1 ± 0.4 μA/cm^2^ was observed between varnish electrode and varnish microflora. Finally, based on Raman and 16S rRNA gene–sequencing results, coculture system of birnessite and *Pseudomonas* (the major Mn oxide and a common electroactive bacterium in varnish) was established to study underlying mechanism. A steadily growing photocurrent (205 μA at 100 h) under light was observed with a stable birnessite after 110 h. However, only 47 μA was generated in the dark control and birnessite was reduced to Mn^2+^ in 13 h, suggesting that birnessite helped deliver electrons instead of serving as an electron acceptor under light. Our study demonstrated that electroactive bacterial communities were positively correlated with Fe/Mn semiconducting minerals in varnish, and diversified EET process occurred on varnish under sunlight. Overall, these phenomena may influence bacterial–community structure in natural environments over time.

## Introduction

Rock varnish, known as “desert varnish,” is a dark–colored Fe/Mn–rich film that forms on rock surfaces in almost every terrestrial weathering environment on earth. On average, rock-varnish thickness may range from 100 μm to several hundred micrometers, with an accumulation rate of 1–15 μm/1000 years (Perry and Adams, 1978; Dorn et al., 1992; Liu and Broecker, 2000; Goldsmith et al., 2012). The elemental composition of rock varnish varies among different rocks, but it commonly comprises clay minerals (70%), amorphous silica and Fe/Mn oxides (about 10–30%) (Potter and Rossman, 1979; Dorn, 2007; Garvie et al., 2008). Rock varnish has been studied by geologists and microbiologists for many years and several theories have been put forward to explain its origin including abiotic origin, biotic processes, or a combination of different mechanisms (Dorn and Oberlander, 1981; Kuhlman et al., 2006; Goldsmith et al., 2014). Notably, varnish is attracting increased attention in the field of astrobiology owing to the recent detection varnish–like geological structures on Mars (Krinsley et al., 2009).

Over the past decades, the microbial diversity of rock varnish in different geographical settings worldwide have gained considerable attention. A diverse microbial ecology of varnish has been analyzed by culture–independent molecular methods from different sites such as the Death Valley, Mojave Desert, Whipple Mountains and Black Canyon (Krumbein and Jens, 1981; Perry et al., 2002; Schelble et al., 2005; Kuhlman et al., 2006, 2008; Northup et al., 2010; Marnocha and Dixon, 2014; Esposito et al., 2015). Despite many published works on mineral composition and microbial diversity of rock varnish all over the world, only a few studies have focus on microbial diversity in Xinjiang, China. Further study is required to understand the microorganisms in these special environments.

Previous studies have focused on the microbe biodiversity within varnish, and Fe/Mn minerals have been reportedly concentrated by bacterial activity, whereas some Mn–oxidizing bacteria have been isolated from varnish (Krumbein and Jens, 1981; Kuhlman et al., 2005; Goldsmith et al., 2014). Although Fe/Mn minerals are used by bacteria as electron acceptors in several metabolic pathways (Lovley, 2006; Weber et al., 2006; Summers et al., 2010; Byrne et al., 2015; Shi et al., 2016), little attention has been given to their semiconducting properties or their influence on bacterial communities. Electron transfer is one of the most fundamental life processes and extracellular electron transfer (EET) in microorganisms is associated with organic matter and elements cycling. Recently research has indicated an unusual interaction between microorganisms and semiconducting minerals under light irradiation. Lu et al. (2012) demonstrated that photoelectrons were produced by the photocatalysis of semiconducting minerals (i.e., rutile, sphalerite, and goethite), and supported the growth of non-phototrophic microorganisms including *Acidithiobacillus ferrooxidans*, heterotrophic *Alcaligenes faecalis*, and a natural soil microbial community. In addition, the non-photosynthetic bacterium *Moorella thermoacetica* was proven to assimilate carbon dioxide into acetate cooperated with the cooperation of cadmium sulfide (CdS) nanoparticles under light illumination (Sakimoto et al., 2016). Although photoenhanced electrochemical interactions have been observed between semiconducting minerals and microorganisms (Feng et al., 2016; Ren et al., 2017, 2018; Zhu et al., 2017). Surprisingly, little attention given to the interaction between Fe/Mn semiconducting minerals and bacterial communities on varnish in natural environments.

To the best of our knowledge, only a few studies have focused on the diverse microbial–community composition of varnish in the Gobi Desert in Xinjiang, China. Thus far, almost no research has analyzed the semiconducting properties of varnish or explored its interaction process under sunlight. The aims of the present study were as follows: (i) to analyze mineral composition through synchrotron radiation X–ray diffraction (SR–XRD), Raman spectroscopy and describe the semiconducting characterization of varnish by electrochemical measurements; (ii) to identify the diversity of bacterial communities from varnish, non-varnish (named substrate) and soil in surrounding environments; (iii) to explore the relationship between semiconducting minerals and microorganisms under visible light and subsequently investigate the complex EET between them. Our results may extend knowledge on mineral–microbe interactions and help elucidate how minerals influence the microbial world especially under sunlight, in natural environments.

## Materials and Methods

### Site Description and Sample Collection

The study area was located at the Desert of Hami, which was a section of the Gobi Desert in Xinjiang, China. Rocks and soil were sampled on June 26, 2017, and the location information were summarized in Table 1. In each site, quintuplicate samples of rock with varnish, non-varnish coated rocks (named substrate) and surrounding soil (the topmost surface soil under sunlight) were collected with a five–point sampling method. All rocks and soil samples were divided into two parts for both microbiological and mineralogical analyses. To assure a sterile condition for molecular analysis, all three kinds of samples were obtained using flame–sterilized tweezers and immediately placed into 1.5 mL sterile tubes contained the first buffer solution of the PowerSoil DNA Isolation Kit (MoBio Laboratories, Carlsbad, CA, United States). The tubes were kept at -20°C before further experiments. In addition, the remaining part of the powder samples was collected sterility and stored at 4°C as sources of inocula for further microbiological experiments. Finally, additional rocks were obtained for laboratory electrochemistry and mineralogy studies.

**Table 1:** Geographic coordinates of sampling sites and altitude above sea level.

Sample number	Sample type	Latitude	Longitude	Elevation (m)
AS	Varnish	43°17′58′′	92°16′52′′	1360
AB	Substrate	43°17′58′′	92°16′52′′	1360
AT	Soil	43°17′58′′	92°16′52′′	1360
BS	Varnish	43°20′55′′	92°12′41′′	1540
BB	Substrate	43°20′55′′	92°12′41′′	1540
BT	Soil	43°20′55′′	92°12′41′′	1540
CS	Varnish	43°00′15′′	93°41′08′′	1070
CB	Substrate	43°00′15′′	93°41′08′′	1070
CT	Soil	43°00′15′′	93°41′08′′	1070
DS	Varnish	43°14′20′′	92°35′22′′	1270
DB	Substrate	43°14′20′′	92°35′22′′	1270
DT	Soil	43°14′20′′	92°35’22′′	1270

### Varnish Mineral Observation and Analysis

To study the morphological features of varnish, rock samples were cut by a diamond saw blade and smoothened with silicon carbide powder. After being impregnated and solidified with polyester resin, the samples were cut into 100–150 μm with wafer cutter and burnished into 30 μm–thick thin sections. These thin sections were observed under an optical microscope (Supplementary Figure S1), and scanning electron microscope (SEM) and the chemical compositions of Fe/Mn mapping were detected by energy dispersive X–ray spectroscopy (EDS). For SEM imaging, the sections were coated with Cr and all above analysis was finished on FEI Quanta 650 FEG system. Then, the varnish mineral composition was investigated by the Synchrotron Radiation X–ray diffraction (SR–XRD) at beamline BL14B1 of the Shanghai Synchrotron Radiation Facility (SSRF) at a wavelength of 0.6887 Å, which was a beamline based on a bending magnet, a Si (111) double crystal monochromator was employed to monochromatize the beam with a focal spot of 0.5 mm (Yang et al., 2015). Furthermore, the Fe/Mn mineral samples were measured by using a Renishaw inVia Reflex system (Wotton–under–Edge, Gloucestershire, United Kingdom) equipped with a 785 nm laser and a long working–distance 50× objective with a focus spot of 1–2 μm and a spectral resolution of 1 cm^-1^. The frequency stability and the accuracy of the apparatus were checked by recording the Raman spectrum of Si. The concentration of Mn^2+^ in the solution was determined via inductively coupled plasma-optical emission spectrometry (ICP-OES, Spectro Blue Sop).

### DNA Extraction, 16S rRNA Amplification and Phylogenetic Analysis

The DNA of samples were extracted with the PowerSoil DNA Isolation Kit, and DNA concentration was determined with a UV–Vis spectrophotometer (Nanodrop ND–1000, United States). The V3–V4 hypervariable regions of bacterial 16S rRNA gene were amplified with the primers 357F and 806R (Huber et al., 2007). For each sample, a 10–digit barcode sequence (provided by Allwegene Company, Beijing, China) was added to the 5′ end of the forward and reverse primers. The PCR was carried out on a Mastercycler Gradient (Eppendorf, Germany) with 50 μL reaction volumes, containing 5 μL 10× Ex Taq Buffer (Mg^2+^ plus), 4 μL 12.5 mM dNTP Mix (each), 1.25 U Ex Taq DNA polymerase, 2 μL template DNA, 200 nM barcoded primers 357F and 806R each, and 36.75 μL ddH_2_O. The cycling parameters were as follows: 94°C for 2 min, 30 cycles of 94°C for 30 s, 57°C for 30 s, and 72°C for 30 s with a final extension at 72°C for 10 min. Three PCR products per sample were pooled to mitigate reaction–level PCR biases. The PCR products were purified by the QIA quick Gel Extraction Kit (QIAGEN, Germany) and quantified via Real Time PCR. Allwegene Company performed the high–throughput paired end Illumina MiSeq sequencing, and high–grade reads were obtained after a complex processing (De Mandal et al., 2016). The sequence reads from the Illumina MiniSeq pyrosequencing of 16S rRNA gene sequences were deposited in the National Center for Biotechnology Information (NCBI), and the Sequence Read Archive (SRA) was under the project accession number of SUB4504299.

### Electrode Fabrication and Electrochemical Measurement

Mineral electrodes were fabricated using varnish, substrate and soil samples brought back from the field. First, three different kinds of samples were ground in a bowl chopper and sieved through a 400–mesh sieve. To prevent shedding, the mineral powder (20 mg) was mixed with anhydrous ethanol (400 μL) and 5% Nafion solution (10 μL). Then, the mixture was dropwise added onto the conductive side of fluorine–doped tin oxide (FTO) electrode. The blank control FTO electrode was dripped with only anhydrous ethanol and Nafion solution. A conventional three electrode system was used (Grätzel, 2001; Hsu et al., 2012), consisting of a mineral electrode, Pt sheet and a saturated calomel electrode (SCE, 0.244 V vs. normal hydrogen electrode) that served as the working, auxiliary and reference electrodes, respectively. Dark and light conditions were realized by an external Light Emitting Diode (LED) having a working wavelength from 400 to 700 nm (Supplementary Figure S2). The light illumination intensity was 100 mW/cm^2^, which was measured by a FGH-1 photosynthetic radiometer (Beijing Normal University Photoelectric Instrument Factory, Beijing, China). Linear sweep voltammetry (LSV) was performed in 0.1 M Na_2_SO_4_ within the potential range from 0 to 1.0 V at a scan rate of 2 mV s^-1^. The photocurrent–time response of the mineral electrodes was determined by an electrochemical workstation (CHI 760E Shanghai Chenhua Instrument, Shanghai, China) at a constant potential of 0.6 V. The measurements were carried out in 0.1 M Na_2_SO_4_ and 0.1 M Na_2_SO_4_ + 1.0 M ethyl alcohol (EA) solution as electrolyte, respectively. All potentials were referenced to the SCE electrode unless otherwise stated in whole paper.

### Bacterial Communities Culture and EET Process Analysis

To gain a better understanding of the EET process that occurred in difference kinds of samples, we took 5 mg of varnish, substrate and soil samples from every four sampling sites. Then, we separately mixed all four varnish, substrate and soil samples. The microbial community from three different kinds of samples were grown in 1/10 diluted Luria–Bertani (LB) medium at 35°C with 200 rpm for 48 h under light irradiation. Subsequently, the cell suspension was inoculated in 2% (v/v) 1/10 diluted LB (35°C, 200 rpm) with agitation until the optical density at 600 nm (OD_600_) reached approximately 1.0. The EETs between minerals and microorganism was investigated through photocurrent–time curves recorded by electrochemical workstation. Photoresponse process for three different mineral electrodes and EET process were measured in a quartz cube cell (10 × 10 × 10 cm) with a conventional three–electrode configuration system (mineral electrode, Pt sheet and a SCE used as working, auxiliary and reference electrode, respectively). Owing to the turbidity for microflora mediums, the actual illumination intensity reached to the electrode surface was approximately 80 mW cm^-2^. The orthogonal experiments for different mediums and mineral electrodes were compared with each other and the electron transferring process should be inferred by the value of light/dark currents.

### Construction of Light–Birnessite–*Pseudomonas* System

Based on the mineral composition and 16S rRNA gene–sequencing results, birnessite (the major Mn oxide) and *Pseudomonas* (a common electroactive bacterium genus present in varnish) were built in a light–Birnessite–*Pseudomonas* system for mechanism study. Considering no pure bacteria were separated from varnish and that *Pseudomonas aeruginosa* is ubiquitously found in natural environments, the pure culture of *P. aeruginosa* PAO1 was selected for the system. Birnessite–type manganese oxide electrodes were prepared by cathodic electrodeposition as reported previously (Ren et al., 2018). *P. aeruginosa* PAO1 was cultured in Luria–Bertani no–sodium (LBNS) medium (Tryptone 10 g L^-1^, Yeast extract 5 g L^-1^) at 35°C with 200 rpm for 24 h and then transferred to the reactor. The reaction system (liquid volume for 120 mL) included a birnessite film coated FTO photoanode, a platinum plate electrode and a SCE. The distance between working electrode and counter electrode was 1 cm. Dark/light cycles were provided by an external LED with an actual illumination intensity, reaching the birnessite electrode surface of approximately 60 mW cm^-2^. The electron transfer process between semiconducting birnessite and *P. aeruginosa* was recorded by a multi–potentiostat (CHI 1000 C, CH Instruments Inc., China) with a potential of 0.6 V. The reactors were placed in a temperature–controlled biochemical incubator (LRH–250, Shanghai, China) with a constant temperature of 35 ± 1°C.

## Results and Discussion

### Mineral Composition of Varnish Samples

**Figure 1:**
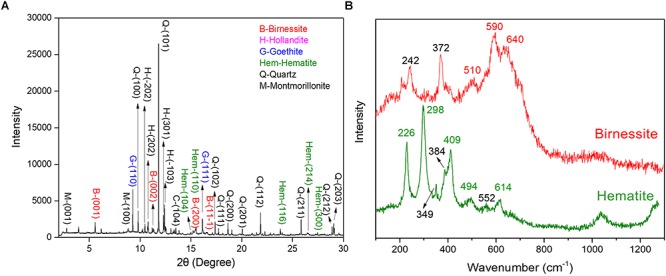
Varnish coated rocks in Xinjiang: **(A)** SR–XRD pattern of minerals composition for rock varnish; **(B)** Raman spectra of mainly Fe/Mn oxides minerals for hematite and birnessite in varnish.

In arid and semiarid China, the surface of Gobi was covered by dense gravel and rock varnish that is ubiquitously distributed (Supplementary Figure S1a). Under optical microscopy, a black to brown colored coating was observed, which was varnish and with a thickness varying from several to hundreds of micrometers. Moreover, the EDS mapping revealed the distribution of Fe/Mn elements (Supplementary Figure S1b). The EDS data revealed that the dominant elements in rock varnish were O, Mn, Fe, Si, and Al. The Mn content and Fe content was 12.42–17.07 and 8.85–11.28 wt%, respectively. Moreover, the O, Si, and Al content were 47.36–50.43, 11.23–16.72, and 6.64–8.78 wt%, respectively. The concentrated Fe/Mn in varnish agreed with the previous results that this thin layer was a manganese–and iron–rich coating and the concentration up to one hundred times higher than that in substrate rocks (Perry and Adams, 1978; Dorn et al., 1992; Thiagarajan and Lee, 2004; Goldsmith et al., 2012). To further explore the mineral composition of varnish, SR–XRD was employed, which had a high signal–to–noise ratio and was suitable for the characterization of nanomaterials and nano–minerals. Figure 1A recorded the XRD patterns, both clay and Fe/Mn minerals were confirmed, such as quartz, montmorillonite, birnessite, hollandite, hematite, and goethite. Considering that Fe/Mn oxides were together with clay minerals and crystallized poorly, their signal was much weaker than of quartz. The main mineral phase of Fe/Mn oxides in varnish, a confocal Raman spectroscopy, was utilized for better understanding. As shown in Figure 1B, the Raman bands at 591 and 641 cm^-1^ were attributed to Mn–O stretching vibration along the chains in MnO_6_ octahedron and the symmetric stretching vibration of MnO_6_ groups, respectively (Julien et al., 2003). The results indicated that the primary Mn oxides were birnessite, which agrees with the previous studies that stated birnessite and birnessite–like minerals are the major phases that occurred in a wide variety of geological settings including soils, desert varnishes, Mn–rich ore deposits and even ocean Mn nodules (Post, 1999). In addition, the primary iron oxides were hematite, which can be identified by two A_1g_ modes (226 and 494 cm^-1^) and three E_g_ modes (298, 409, and 614 cm^-1^) (De Faria et al., 1997). Based on these results we concluded that Fe/Mn was enriched in varnish, and the major mineral phases were hematite and birnessite.

### Photo-Response of Varnish and Semiconducting Properties

**Figure 2:**
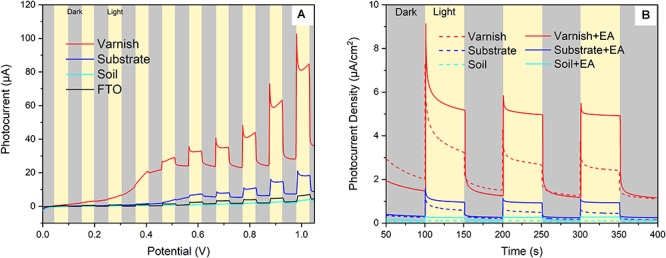
Visible light response of mineral electrodes **(A)** Linear sweep voltammetry results of the mineral electrodes in dark/light conditions; **(B)** Photocurrent–time behaviors of different mineral electrodes (varnish, substrate, and soil) in supporting electrolyte of 0.1 M Na_2_SO_4_ with and without the presence of ethyl alcohol (EA).

Hundreds of semiconducting minerals are found on Earth such as Fe/Mn oxides (e.g., hematite, pyrolusite), Ti/Ti–Fe oxides (e.g., rutile and ilmenite), and sulfides (e.g., sphalerite, pyrite) (Xu and Schoonen, 2000; Lu et al., 2012). The electronic structure of semiconducting minerals can often be characterized by a filled valence band (VB) and an empty conduction band (CB). When energy was absorbed, electrons in the VB can be excited to the CB. This process leads to the separation of electron–holes, which can induce redox reactions (Grätzel, 2001). In order to demonstrate the semiconducting properties of varnish and examine their response to sunlight, mineral electrodes were fabricated with real field varnish, substrate and soil samples. Subsequently, their photocurrent output diversity under light irradiation (illumination intensity of 100 mW/cm^2^) were contrasted. The dark/light linear sweep voltammetry (LSV) curves (Figure 2A) showed that both soil and FTO electrodes produced negligible dark currents, varnish and substrate electrode had an average current of 24.5 ± 4.2 and 6.2 ± 1.5 μA, respectively. Upon light illumination, all photocurrents increased in varying degrees. Notably, the photocurrent for varnish electrode was as high as 32.7 μA at 0.6 V and even reached to 82.4 μA at 1.0 V. However, the photocurrents for other electrodes were lower than 20 μA at 1.0 V. The results indicated that varnish had good response to solar light and its remarkable photocurrent was attributed to the photocatalytic reaction.

Furthermore, the repeatability of light–response for mineral electrodes was investigated by using photocurrent–time curves. As shown in Figure 2B, the photocurrent density of varnish electrode was 3.1 ± 0.4 μA/cm^2^ under light illumination, which was almost 6 times higher than that of the value of the control substrate samples (0.5 ± 0.2 μA/cm^2^) under the same conditions. Notably, the photocurrent response of varnish mineral electrode was further enhanced to 5.1 ± 0.2 μA/cm^2^, owing to the suppressed direct charge carrier recombination when EA was utilized as hole scavengers in electrolyte. The anodic photocurrents corresponding to the photo–oxidation process indicated that varnish had an n–type (mostly charge carriers were free electrons) semiconducting nature which was in accord with the fact of n–type for hematite and birnessite (Xu and Schoonen, 2000; Hsu et al., 2012; Ren et al., 2017). These six–time higher photocurrents for varnish than substrate minerals demonstrated that rock varnish exhibited photoelectrochemical activity in response to light, and its semiconducting properties should be associated with the photogenerated electron–transfer process and maybe had potential influencing biocatalysis metabolism in natural conditions.

### General Characteristics of Bacterial Communities in Different Samples

**Figure 3:**
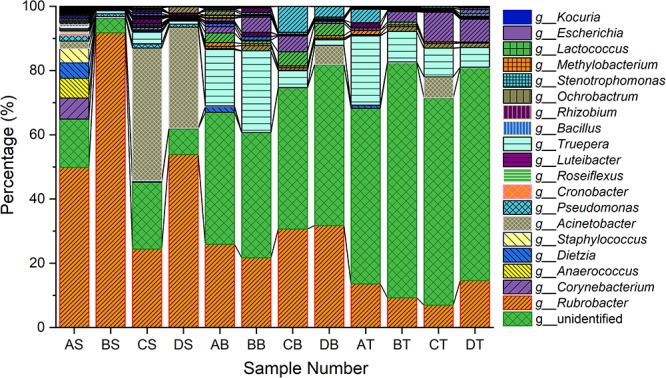
Taxonomy classifications of OTUs at class levels for three kinds of samples (Only top 10 enriched class 259 categories were shown in the figure). AS, BS, CS, DS were the varnish samples from four different regions, AB, BB, CB, DB and AT, BT, CT, DT represented the substrate and soil samples, respectively.

The bacterial community in varnish, substrate and soil samples was investigated via High–throughput Illumina sequencing technique. The flat extent of the rarefaction curves indicated that the major parts of the bacterial communities in all samples were covered (Supplementary Figure S3). After quality and chimera evaluation, a total of 378367 final tags and 5262 operational taxonomic units (OTUs) were obtained from 12 samples. Moreover, 1249, 1629, and 2384 OTUs were identified as belonging to varnish, substrate and soil samples, respectively. All soil samples exhibited higher variety than the other two kind of sample which is in accordance with the Observed species and Goods coverage results (Supplementary Table S1). The varnish samples have lower diversity than the substrate and soil samples as they showed a lower Shannon result and Chao1 index. In addition, 15 phyla were present across the 12 samples, of which 10 phyla accounted for almost 95%. *Actinobacteria* dominated all samples with average percentages of 35 to 94% (Supplementary Figure S4). The phyla present in the substrate and soil samples were similar to one another, and they differed from the varnish samples (Figure 3). At the genus level, *Rubrobacter* accounts for a large proportion (more than 30%) at each sample, which belongs to *Actinobacteria* class, and is thought to be a radiation resistant bacterium (Terato et al., 1999). This bacterium had been isolated from various extreme conditions, such as hot spring, Gobi Desert (Suzuki et al., 1988; Zhang et al., 2012). Previous studies indicated that some isolated bacteria are capable of oxidizing or reducing Mn (Dorn and Oberlander, 1981). Mn oxidizing/reducing bacteria were identified in our study, such as *Acinetobacter, Pseudomonas, Bacillus, Rhizobium*, and *Brevundimonas.* The relationship between these bacteria and Fe/Mn minerals was familiar with us. However, it should be point out that these oxidizing or reducing metabolism process were closely related to the EET process by microorganisms.

### Microbial Community Structure Characteristics and Cluster Analysis

**Figure 4:**
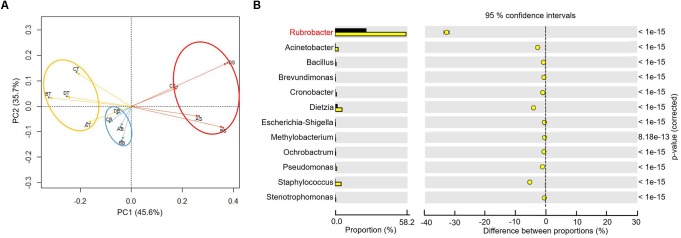
**(A)** The PCA based on the relative OTUs abundance indicated that three kinds of samples with different covering formed distinct communities (red–varnish; blue–substrate; orange–soil); **(B)** Statistical analysis of microbiology on the surface (the yellow point) and substrate (the black point), the *p*-value was less than 0.01.

To better understand the microbial–community structure and its relationship with minerals. Principal components analysis (PCA) was performed to determine the relative abundance of OTUs, which can simplify complex problems and is usually used to analyze the influencing factors from polyelement. As shown in Figure 4A, the PCA results indicated that the varnish, substrate and soil samples had different coverings and formed distinct communities. In addition, all three kinds of samples clustered together, respectively, and the distance between the soils and substrates was smaller than that with varnish samples, suggesting that the bacterial communities in varnish were highly different with the others. We calculated the PCA values to identify the major microorganisms that contribute to the PCA results. An interesting phenomenon was observed, in which 11 electroactive microorganisms appeared in varnish, contributing significantly to the PCA results (Table 2). The *t*-test statistical analysis was performed to further explore the distribution variations of the 11 electroactive microorganisms, and the results repeatedly showed that these electroactive microorganisms gathered on varnish (Figure 4B).

**Table 2:** Electroactive genera in alphabetical order and the references for electroactivity.

No.	Genera	PC1(%)	Reference
1	*Acinetobacter*	26.482	Erable et al., 2010
2	*Bacillus*	24.465	Nimje et al., 2009; Cournet et al., 2010
3	*Brevundimonas*	32.975	Cournet et al., 2010
4	*Cronobacter*	23.913	Rezaei et al., 2009
5	*Dietzia*	24.363	Sacco et al., 2017
6	*Escherichia*	38.44	Ahmed et al., 2014
7	*Methylobacterium*	29.139	Reda et al., 2008
8	*Ochrobactrum*	27.796	Zuo et al., 2008
9	*Pseudomonas*	33.218	Su et al., 2012
10	*Staphylococcus*	27.873	Cournet et al., 2010
11	*Stenotrophomonas*	33.812	Linke et al., 2017

To gain more insight, we calculated the total number of electroactive genera varnish, substrate and soil samples. All the four sample sites showed that the electroactive microorganisms gathered on varnish and fewer appeared in the other two kinds of samples (Table 3). One key question raised was why electroactive microorganisms gathered on varnish. Based on mineral analysis and photoelectrochemical measurements, we concluded that varnish is a thin coating enriched with semiconducting Fe/Mn oxides. The negatively charged electrons and positively charged holes in the CB and VB can be yielded after absorbing light energy. These electron–hole pairs then induced redox reactions, and the semiconducting minerals can serve as electron conduits for different redox reactions. Previous studies have demonstrated that the photo-holes can combine with the electrons from microorganisms, and the photoenhanced electrochemical interaction occurred between hematite and *Shewanella, Geobacter*, or even the bacterial community under light (Feng et al., 2016; Ren et al., 2017; Zhu et al., 2017). Thus, other issues have emerged. First, do these electroactive microorganisms have a relationship with the semiconducting Fe/Mn minerals on varnish? Second, can the semiconducting minerals in varnish participate in the EET process? These semiconducting Fe/Mn oxide minerals in varnish, may possibly have a long–existing pathway, participating in the EET process and influencing bacterial–community structure in local environments, but they are not given much attention.

**Table 3:** Total final tags and relative abundance for electroactive genera in all samples.

	Site A		Site B		Site C		Site D	
Varnish (S)	3679	16.34%	904	2.17%	8560	42.95%	10958	32.87%
Substrate (B)	618	2.21%	40	0.13%	61	0.18%	1970	5.90%
Soil (T)	394	1.13%	33	0.11%	2280	6.44%	63	0.18%

### EET Process Between Semiconducting Minerals and Bacterial Communities

**Figure 5:**
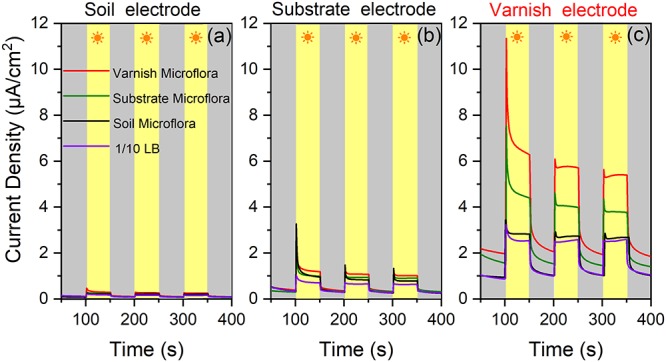
**(a)** Current densities between soil mineral electrode and four kinds of microflora solution mediums (bacterial communities from varnish, substrate, soil, and blank 1/10 LB) under light and dark conditions; **(b)** Current densities between substrate rock electrode and the four kinds of microflora solution; **(c)** Current densities between varnish mineral electrode and different mediums.

The *in situ* currents between electroactive microorganisms and semiconducting minerals on varnish are difficult to measure. However, these currents should be observed after culturing the bacterial communities. Undoubtedly, primitive microbial communities change once cultured, but different currents may be obtained between mineral electrodes and the cultured microorganisms owing to the numerous electroactive genera that gathered on varnish. It would be possible for observing the EET process occurred between semiconducting minerals and “electroactive genera” from varnish under the same culture condition. Accordingly, the bacterial communities from varnish, substrate and soil samples were cultured by 1/10 LB under light irradiation at the same time. The EET between mineral electrodes (varnish, substrate, and soil) and these microfloras were explored in detail under light and dark conditions. As shown in Figure 5, significant currents can be observed only when varnish electrode was employed. When using electrodes made by substrate or soil samples, the photocurrents were negligibly, and their average value was lower than 1 and 0.2 μA/cm^2^, respectively. These slight currents should be ascribed to the lower concentration of Fe/Mn semiconducting minerals in substrate or soil than that in varnish, which was consistent with the LSV results in Figure 2.

Comparison of the currents between the varnish electrode and the four kinds of mediums (bacterial communities from varnish, substrate, soil and blank 1/10 LB), showed that they significantly differed, which can be due to the microflora media. Notably, the most remarkable photocurrent was observed between the varnish electrode and the varnish microflora, with the average value reaching 6.1 ± 0.4 μA/cm^2^. However, the values of photocurrents in the substrate and soil microflora were 3.9 ± 0.2 and 2.9 ± 0.1 μA/cm^2^, respectively, all of which were higher than that in 1/10 LB medium (2.4 ± 0.1 μA/cm^2^) (Figure 5). In addition, bacterial community studies indicated that the percentage of electroactive genera in varnish, substrate and soil microflora were 62, 43, and 38%, respectively (Supplementary Figure S5). The absolute growth of the photocurrents indicated that a high number of electroactive compounds appeared in varnish culture, which was possibly associated with the electroactive genera in varnish. Moreover, when using a substrate electrode or a soil electrode, the photocurrents decreased in the order varnish microflora > substrate microflora > soil microflora (red > green > black). These results showed a good electron transfer process between mineral electrodes and varnish-cultured microflora under light irradiation, indicating that the EET between semiconducting minerals and electroactive bacterial communities may have occurred on the varnish under sunlight in a natural environment.

### Mechanism Study Based on “Light–Birnessite–*Pseudomonas aeruginosa*”

**Figure 6:**
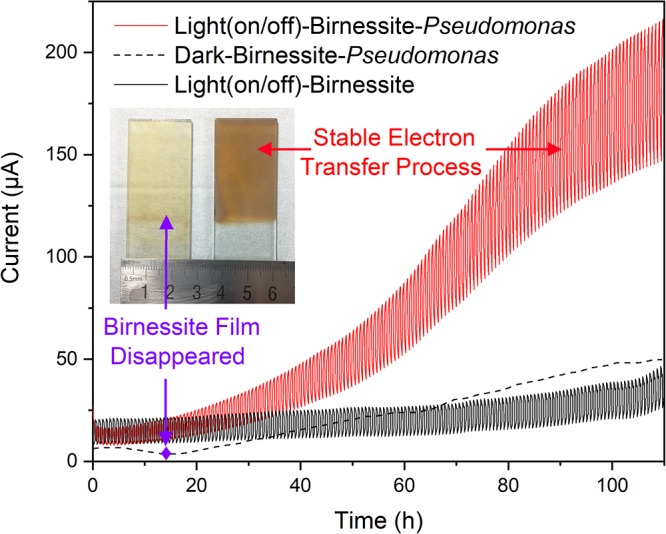
Currents comparison for “light–birnessite–*Pseudomonas*” system under dark/light illumination cycles (Insert picture: Morphology of birnessite electrodes after used in different systems).

To clarify the detailed EET process and understand the interaction mechanism between semiconducting minerals and microorganisms in varnish under light, we further built a pure bacteria culture system and explored its performance. Based on the mineral and bacterial community analysis results in varnish. Birnessite, a major mineral phase of Mn oxide in desert varnishes, and *P. aeruginosa*, an electrochemical activity bacterium ubiquitous found in environments (actual average value of relative abundance in varnish, substrate and soil samples: 1.85, 0.02, and 0.02%, respectively) were chosen. The photocurrent results for “Light–Birnessite–*Pseudomonas*” system with a reduplicative 1200 s dark/light illumination cycles (laboratory-based simulated day-night cycles) are presented at Figure 6. In the negative control without *Pseudomonas*, the photocurrents were stable and with an average value of 25 μA. Notably, in the “Light (on/off)–Birnessite–*Pseudomonas*” system, a steadily growing current generated, both photo/dark currents increased at a higher speed after 40 h and their value reached at 205 and 137 μA at 100 h, respectively. The enhanced photocurrents were ascribed to *Pseudomonas*, indicating semiconducting birnessite transferred out more electrons from *P. aeruginosa* PAO1 under light condition. This result demonstrated that the enhanced EET process was realized with the cooperation of *P. aeruginosa* and semiconducting birnessite under light illumination.

In order to further understand the interactions in the “light-mineral–microbe” system, a “Dark–Birnessite–*Pseudomonas*” control was conducted. Notably, the current was only 47 μA at 100 h, which was considerably lower than that in the light system. The birnessite film was reduced to Mn^2+^ (0.21 μM) after 13 h as shown in Figure 6 (insert picture), suggesting that birnessite played a role of electron acceptor. Whereas, the birnessite electrode can remain stable even after 110 h in “Light (on/off)–Birnessite–*Pseudomonas*” system, suggesting that birnessite did not act as an electron acceptor. Under light, the semiconducting property of birnessite was activated and photoelectron–holes were generated at the same time, leading to mineral photocatalysis. The photoexcited holes had more positive potential and can easily combine with the electrons produced by microorganism metabolisms. High currents were maintained at the light on/off system, indicating that the efficient electron transfer process occurred on the surface of birnessite and mineral structure was “protected” after harvesting light energy. Hence, the electron–transfer rate changed drastically, and photocurrents steadily increased over time.

### EET Possibly Occurred on Varnish Under Natural Light Conditions

Manganese and Iron are common variable valence elements in nature and the Fe/Mn oxides or oxyhydroxides are widely distributed throughout the earth environments (Koschinsky and Halbach, 1995; Boston et al., 2008). Previous studies have indicated that Fe/Mn oxides are closely related to the metabolic processes of microorganisms, especially Fe/Mn oxidizing bacteria (Emerson et al., 2010), and these Fe/Mn oxides could protect microorganisms from ultraviolet radiation (Friedmann, 1982; Hughes and Lawley, 2003). However, limited attention has been devoted to the semiconducting properties of these Fe/Mn oxides in nature and little attention focus on their interaction with bacteria after sunlight activation. In recent years, research on the electron transfer between electricigens and semiconducting materials under light illumination has notably progressed (Lu et al., 2012; Sakimoto et al., 2016). Our research demonstrated that both electroactive microorganisms and semiconducting Fe/Mn oxides gathered on varnish. When these minerals are exposed to sunlight, the photoelectron holes could be produced and the EET process should be exist on varnish for a long geological history.

**Figure 7:**
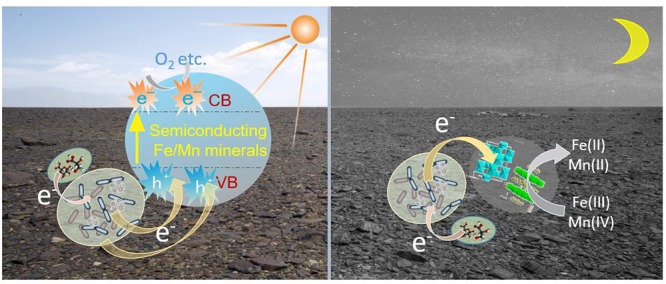
Schematic diagram of possible EET process between microorganisms and Fe/Mn semiconducting minerals occurred on varnish at day and night times.

Fe/Mn oxides are abundant on the Earth’s surface and serve as the most common natural electron acceptors for EET (Shi et al., 2016). However, under sunlight irradiation in daytime, semiconducting minerals should be stimulated, generating photoelectron–holes, and a rapid electron transfer process should occur on the varnish as shown in Figure 7. Owing to the semiconducting properties, photo-excited holes combined with electrons from microorganisms. Therefore, Fe/Mn oxide no longer acted as an electron acceptor but took part in the EET process, delivering them to surrounding environments. At night or during the absence of sunlight, the oxidation of organic matter is realized duo to microbial metabolism, in which Fe/Mn oxides serve as electron acceptors (Weber et al., 2006; Shi et al., 2012). From an ecological perspective, EET is an efficient way to cope with the limitations in habitat for electroactive microorganisms (Klüpfel et al., 2014; Koch and Harnisch, 2016). With the cooperation of semiconducting minerals and sunlight, a local field effect is generated, resulting in more efficiently electron transfer process, and this phenomenon affects the structures of bacterial communities and facilitate electroactive microorganism accumulating on varnish over time. Notably, both electroactive genera and Fe/Mn semiconducting minerals are positively correlated with the EET process under light. Further studies are warranted to understand EET in natural environments, especially under sunlight irradiation in the future.

## Conclusion

Rock varnish is commonly found on rock surfaces throughout the arid regions of the world. In this study, we analyzed the mineral composition and bacterial communities of varnish in Xinjiang. Electrochemical measurements demonstrated that varnish had photoelectrochemical activity in response to visible light owing to the semiconducting Fe/Mn oxides. The bacterial community for varnish, substrate and surrounding soil were analyzed, and the PCA results indicated that electroactive microorganisms gathered on varnish. Orthogonal experiments for EET between mineral electrodes and cultured microflora and pure cocultured system of birnessite and *Pseudomonas* demonstrated that efficient EET process can occur on varnish under light. The electroactive bacterial communities were positively correlated with Fe/Mn oxides in varnish, and the bacterial–community structure was influenced by semiconducting minerals. All of these findings presented a greater possibility for electron flow under sunlight in natural environments.

## Author Contributions

GR and HD designed the experiments. GR and YY carried out the experiments. GR, YY, YN, and HD analyzed the experimental results. GR wrote the manuscript. HD, XW, and AL performed revisions. GR, HD, YL, and CW analyzed the data in the Supplementary Materials and revised them. AL, YL, and CW funded the study. All authors agree to submit the work to Frontiers in Microbiology and approved it for publication.

## Conflict of Interest Statement

The authors declare that the research was conducted in the absence of any commercial or financial relationships that could be construed as a potential conflict of interest.
