# Supplementary material for: Natural Extracellular Electron Transfer Between Semiconducting Minerals and Electroactive Bacterial Communities Occurred on the Rock Varnish
Source: Front Microbiol. 2019 Mar 4;10:293. doi: 10.3389/fmicb.2019.00293 (PMC6410676; doi:10.3389/fmicb.2019.00293)
Supplement: Supplementary file 1 [file Table_1.DOCX]

Supplementary Material

Natural Extracellular Electron Transfer Between Semiconducting Minerals and Electroactive Bacterial Communities Occurred on the Rock Varnish

Guiping Ren^1^, Yingchun Yan^2^, Yong Nie^2^, Anhuai Lu^1*^, Xiaolei Wu^2^, Yan Li^1^, Changqiu Wang^1^, Hongrui Ding^1*^

^1^ The Key Laboratory of Orogenic Belts and Crustal Evolution, Beijing Key Laboratory of Mineral Environmental Function, School of Earth and Space Sciences, Peking University, Beijing, China;

^2^ College of Engineering, Peking University, Beijing, China

*** Correspondence:**Anhuai Lu. E-mail: ahlu@pku.edu.cn or to Hongrui Ding. E-mail: DHR@ pku.edu.cn

**
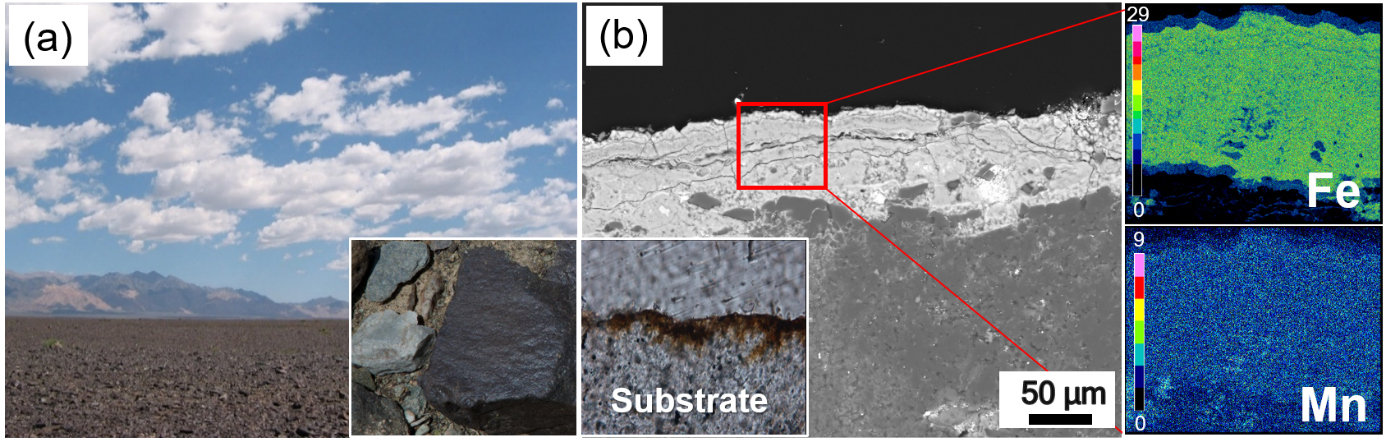
**

**Figure S1.** Varnish coated rocks in Xinjiang: (a) Photographs from field and hand specimen (Insert picture); (b) Micrograph of varnish showed by SEM and EDS mapping of Fe/Mn elements (Insert: optical micrograph).


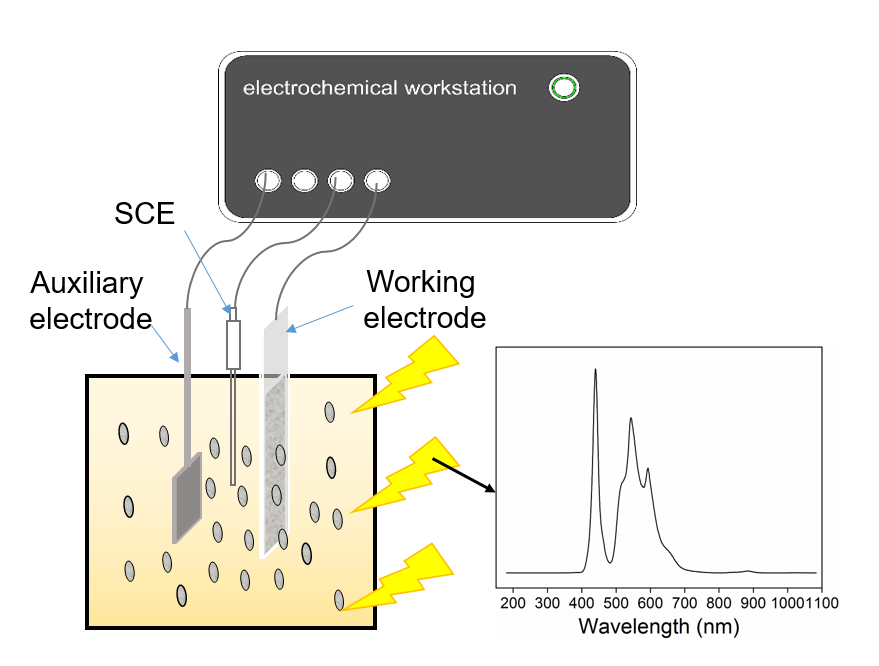


**Figure S2.** The conventional three–electrode configuration system and the working wavelength of LED utilized in experiments.


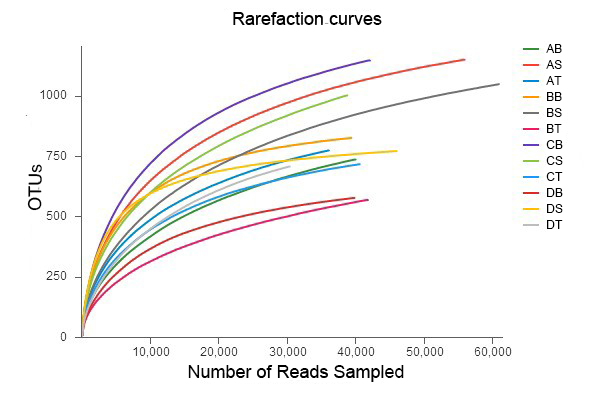


**Figure S3.** Rarefaction curves reach saturation for all samples, a tending trend meaning that no further sampling depth is needed to accumulate diversity.

**Table S1.** List of Final tags, operational taxonomic units (OTUs), and microbial community diversity indicts. Final tags were obtained after a series of quality control with mothur. Chao1 was the species richness index, which was used to estimate the number of OTU in the community. Observed species was the number of OTU that actually observed with the increase of sequencing depth. Shannon was used to estimation of microbial diversity index in samples. For Observed species and Goods coverage were used to assessment the quality of sequencing.

| Samples | Final tags | OTUs | Chao1 | Shannon | Observed species | Goods coverage |
| --- | --- | --- | --- | --- | --- | --- |
| AS | 22519 | 183 | 218.21381 | 4.0524299 | 172.1 | 0.9974762 |
| AB | 28026 | 379 | 417.99115 | 5.6988901 | 337.4 | 0.9951188 |
| AT | 34838 | 470 | 491.51391 | 6.0883409 | 412.4 | 0.9942577 |
| BS | 41575 | 326 | 342.36694 | 1.9058926 | 253.5 | 0.9945903 |
| BB | 31396 | 396 | 440.30928 | 5.2576302 | 336.3 | 0.9944537 |
| BT | 29308 | 688 | 725.25453 | 7.3557570 | 630.2 | 0.9930879 |
| CS | 19932 | 371 | 409.41826 | 4.2690965 | 360.4 | 0.9956354 |
| CB | 32999 | 415 | 450.98949 | 5.8537970 | 354.2 | 0.9941746 |
| CT | 35404 | 602 | 633.83932 | 6.4309485 | 515.1 | 0.9928860 |
| DS | 33336 | 369 | 396.26682 | 3.1201738 | 301.0 | 0.9909846 |
| DB | 33366 | 439 | 474.72368 | 5.4031494 | 372.2 | 0.9940914 |
| DT | 35668 | 624 | 655.99108 | 6.8909757 | 572.8 | 0.9930997 |





**Figure S4.** Taxonomy classifications of OTUs at phyla levels for three kinds of samples. (Only top 10 enriched class categories are shown in the figure).





**Figure S5.** The percentage of electroactive genera in varnish, substrate and soil microflora 62%, 43% and 38%, respectively.
